# Conditional over-expression of RAGE by embryonic alveolar epithelium compromises the respiratory membrane and impairs endothelial cell differentiation

**DOI:** 10.1186/1465-9921-14-108

**Published:** 2013-10-17

**Authors:** Duane R Winden, Nicholas T Ferguson, Benjamin R Bukey, Alexander J Geyer, Alex J Wright, Zac R Jergensen, Adam B Robinson, Jeffrey A Stogsdill, Paul R Reynolds

**Affiliations:** 1 Department of Physiology and Developmental Biology, Brigham Young University, 375A Widtsoe Building, 84602 Provo, UT, USA

**Keywords:** RAGE, Transgenic, Endothelium, Lung, Collagen

## Abstract

**Background:**

Receptors for advanced glycation end-products (RAGE) are cell surface receptors prominently expressed by lung epithelium. Previous research demonstrated that over-expression of RAGE by murine alveolar epithelial cells during embryogenesis caused severe lung hypoplasia and neonatal lethality. However, the effects of RAGE over-expression on adjacent matrix and endothelial cells remained unknown.

**Methods:**

RAGE transgenic (TG) mice were generated that conditionally over-expressed RAGE in alveolar type II cells when fed doxycycline (dox) from conception to E18.5. To evaluate effects on the basement membrane, immunostaining and immunoblotting were performed for collagen IV and MMP-9, a matrix metalloprotease capable of degrading basement membranes. To assess changes in vasculature, immunostaining, immunoblotting and qRT-PCR were performed for Pecam-1, a platelet endothelial cell adhesion marker also known as CD31. Lastly, to characterize potential regulatory mechanisms of endothelial cell differentiation, immunoblotting and qRT-PCR for FoxM1, a key endothelium-specific transcription factor of the Forkhead Box (Fox) family, were completed.

**Results:**

Qualitative immunostaining for collagen IV was less in RAGE TG mice compared to controls and immunoblotting revealed decreased collagen IV in the RAGE TG mouse lung. Additionally, elevated MMP-9 detected via immunostaining and immunoblotting implicated MMP-9 as a possible down stream effector in matrix destabilization mediated by RAGE signaling. Lastly, Pecam-1 assessment revealed a decrease in the prevalence of microvascular endothelial cells coincident with FoxM1 abrogation in RAGE TG mice compared to controls.

**Conclusions:**

RAGE over-expression by alveolar epithelium weakened the basement membrane and associated matrix via increased MMP-9 activity. Furthermore, over-expression of RAGE inhibited FoxM1, suggesting that anomalous transcriptional control contributes to decreased endothelial cell prevalence in the TG mouse lung.

## Background

Lung development results from reciprocal interactions between respiratory epithelium derived from the foregut endoderm and the surrounding splanchnic mesenchyme. A sequential series of developmental events leads to the formation of airways juxtaposed with vast capillary networks necessary for efficient gas exchange in the neonate [1]. Each of these programmed events reveals that normal pulmonary morphogenesis depends on a very specific program of temporal and spatial control of cellular differentiation, migration, and proliferation.

The receptor for advanced glycation end products (RAGE) is a cell-surface membrane protein of the immunoglobulin superfamily composed of three domains: an extracellular ligand binding domain, a domain necessary for membrane docking, and a cytosolic domain essential in the perpetuation of intracellular signaling events [2]. RAGE is basally expressed in diverse locations throughout the body and it is most abundantly expressed by lung alveolar epithelium [3]. While its role in development is less understood, RAGE has been implicated in cell spreading and adherence [4]. Demling et al. specifically showed that RAGE is localized to the baso-lateral membrane of human alveolar epithelial cells, and that increased expression of RAGE in these cells leads to increased binding to collagen and a squamous cellular morphology [4]. Another plausible role for RAGE in development may involve its ability to control apoptosis that refines epithelial cell quantity in definitive alveoli [5].

While the precise control of RAGE expression during lung organogenesis likely assists in controlling lung-specific genetic programs, its participation in lung inflammatory signaling in the adult may explain developmental abnormalities when expression levels rise. RAGE is already widely known to bind advanced glycation end-products (AGEs) during the orchestration of vascular inflammation related to hyperglycemia [6]; however, the impact of elevated receptor availability on alveolar vasculature during embryogenesis has not been clearly tested. In addition to AGEs, RAGE also binds cytokine-like mediators of the S100/calgranulin family, high mobility group box 1 (HMGB-1) and amyloid β-fibrils [2,7]. These ligands further implicate downstream signaling pathways potentially involved in mechanisms of abnormal lung derivation. For example, S100s and HMGB-1 lead to RAGE-mediated activation of Ras and NF-kB [8,9]. Because these signaling molecules increase in cases of elevated apoptosis and matrix resorption, RAGE may initiate a signaling axis wherein embryonic tissue loss and irreversible parenchymal remodeling occur.

Previous research showed that a conditional transgenic (TG) mouse model that enhances RAGE expression exhibited neonatal lethality due to severe pulmonary hypoplasia [10]. In the current study, the effect of RAGE over-expression on the development of the alveolar basement membrane and adjacent vasculature were analyzed. Electron microscopy of TG lungs identified notable separation of ATI cells from fragmented basement membranes. The current research suggests that RAGE over-expression causes deterioration of the alveolar basement membrane through MMP-9 mediated collagen IV destruction, and that RAGE over-expression diminishes vascularization during organogenesis, possibly via inhibition of FoxM1.

## Methods

### Animals

All mice were in a C57Bl/6 background. RAGE Transgenic (TG) mice that overexpress RAGE were created by mating *SP-C*-rtTA and *TetO*-RAGE single transgenic mice and transgene expression was induced by feeding dams doxycycline (dox) from before conception until sacrifice date at E18.5 [10]. Animal use was in accordance with IACUC protocols approved by Brigham Young University.

### Lung morphology and immunohistochemistry

Lungs were fixed in 4% paraformaldehyde, embedded in paraffin, and 5 μm sections were obtained. Standard H&E staining was performed and immunohistochemistry was conducted using standard procedures previously elaborated [11]. Sections were dehydrated, deparaffinized, and antigen retrieval was performed using the citrate buffer method [12,13]. Slides were blocked, incubated with primary and appropriate secondary antibodies that utilize HRP conjugation with the Vector Elite Kit (Vector Laboratories; Burlingame, CA). Antibodies for the following were used: Pecam-1 (1:500, BD Pharmingen, San Jose, CA, #553370), collagen IV (1:500, Abcam, Cambridge, MA, ab6586), and MMP-9 (1:200, Santa Cruz Biotechnology, Santa Cruz, CA, sc-6840). No staining was observed in sections without primary or secondary antibody. Picro-sirius red staining was performed to assess general collagen (Direct Red 80, Sigma-Aldrich, Saint Louis, MO, #365548).

### Electron microscopy

Whole mouse lungs were excised and fixed overnight in a 2% glutaraldehyde/0.06 M sodium cacodylate solution (pH 7.3), rinsed in sodium cacodylate and placed in 1% osmium tetroxide/sodium cacodylate, then uranyl acetate overnight. Tissues were dehydrated in a graded acetone series and embedded in Spurr’s resin. 80 nm sections were obtained using a RMC MT-X Ultra Ultramicrotome (Tucson, AZ) and stained using Reynolds’ lead citrate. Sections were photographed using a FEI Tecnai T12 electron microscope.

### Immunoblotting

Lungs from E18.5 mouse embryos were homogenized in RIPA buffer with protease inhibitors (Thermo Fisher). BCA quantification was performed to ensure equal sample loading (Thermo Fisher). Immunoblotting was performed using antibodies against Pecam-1 (Santa Cruz, 1:1,000, sc-376764), FoxM1, (Santa Cruz, 1:1,000, sc-271746), collagen IV (Abcam, 1:5,000, ab6586) and MMP-9 (Santa Cruz, 1:500, sc-6840) using standard protocols discussed in previous work [8,14]. Goat anti-rabbit (Vector Labs, Burlingame, CA, PI-1000 secondary antibody concentration was 1:10,000 for collagen IV and 1:5,000 for all other blots. Band densities were assessed using UN-SCAN-IT software (Silk Scientific, Orem, UT).

### qRT-PCR

Quantitative Real-Time PCR was performed using total RNA from lungs of E18.5 mice, and was performed as previously described [15]. After isolation, total RNA was converted to cDNA, and qRT-PCR was performed using primers specific for *Pecam-1* (5′-CTCCAACAGAGCCAGCAGTA-3′ and 5′-GACCACTCCAATGACAACCA-3′), *FoxM1* (5′-GCGACTCTCGAGCATGGAGAATTGTCACCTG-3′ and 5′-GCGCTACTCGAGTTCGGTTTTGATGGT-3′), and *GAPDH* (5′-CAGGGCTGCTTTTAACTCTGG-3′ and 5′-TGGGTGGAATCATATTGGAACA-3′) synthesized and HPLC purified by Invitrogen Life Technologies (Grand Island, NY).

### Statistical analysis

Results are presented as the means ± S.D. of six replicate pools per group. Means were assessed by one and two-way analysis of variance (ANOVA). When ANOVA indicated significant differences, student t tests were used with Bonferroni correction for multiple comparisons. Results are representative and those with p values <0.05 were considered significant.

## Results

### Embryonic up-regulation of RAGE destabilizes the respiratory membrane

**Figure 1:**
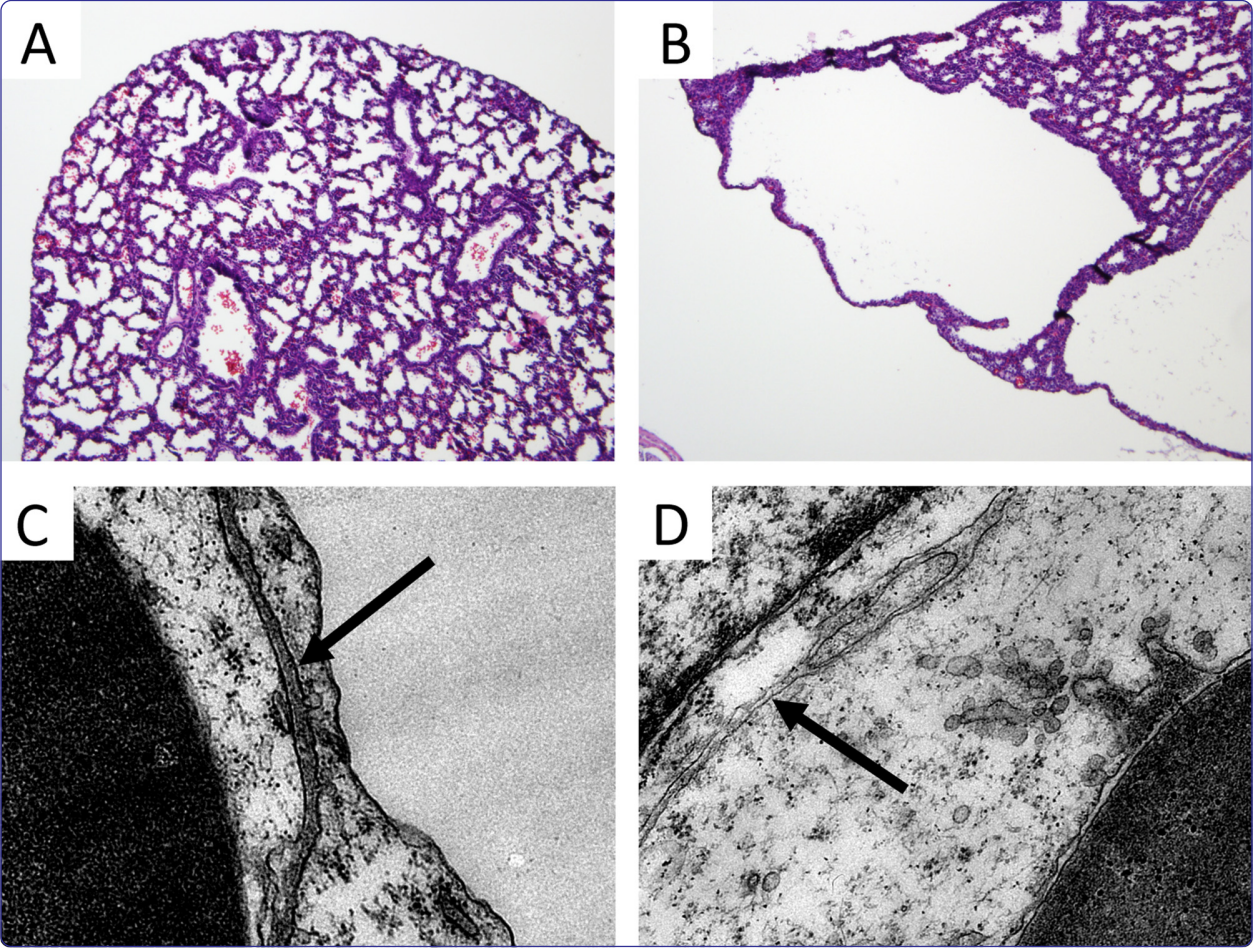
**Lung histology and ultrastructure in RAGE TG mice compared to controls.** Significant lung hypoplasia was observed in RAGE TG mice exposed to doxycycline from conception to E18.5 **(B)** compared to lungs from control littermates **(A)**. Images were at 100X original magnification. Electron microscopy of RAGE TG and control mice fed doxycycline from conception until sacrifice date at E18.5 revealed altered basement membrane (BM) integrity in the alveolar compartment. Wild type BM was distinct and pronounced **(C**, arrow**)** compared to RAGE TG BM that appeared diminished and fragmented **(D**, arrow**)**. Magnification for each image is 6,500X.

Traditional staining with hematoxylin and eosin revealed a striking phenotype wherein the distal lung architecture in RAGE TG mice lacked sufficient parenchymal tissue (Figure 1A and B). Isolated areas of distal lung development were sporadically observed; however, vacuous regions that lacked matrix and associated cells were common. A closer inspection of RAGE TG lung tissues by electron microscopy identified diminished, poorly fused basement membranes that comprise the respiratory membrane (Figure 1C and D, arrows). Because of significant lung tissue loss and only diffuse basement membranes in the lungs of E18.5 RAGE TG mice following uninterrupted dox administration, our focus centered on basement membrane stability in the context of RAGE up-regulation.

### Collagen is diminished in RAGE over-expressing lungs

**Figure 2:**
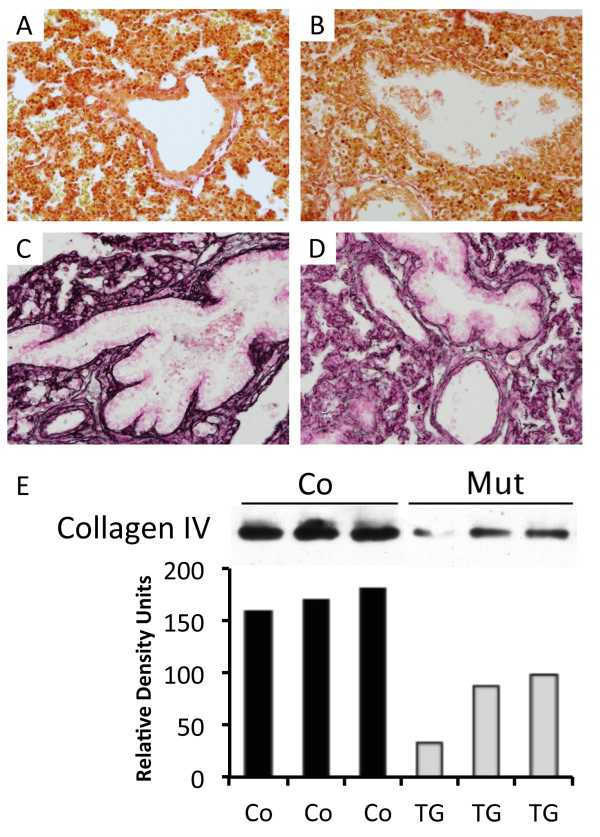
**Collagen expression in RAGE TG mice compared to controls.** Lungs from Control **(**Co, **A** and **C)** and RAGE TG **(B** and **D)** mice were stained for Pico-Red **(A** and **B)** to visualize total collagen content and type IV collagen **(C** and **D)** and only qualitative decreases were observed. Images were at 100X original magnification. Immunoblotting for type IV collagen using equal aliquots of 10 μg total lung protein revealed markedly decreased total type IV collagen expression in RAGE TG mouse lungs compared to controls **(E)**. *Statistical difference (P ≤ 0.05) with at least three replicates per group.

A general analysis of collagen was completed using a picro-sirius red stain, and the data revealed little qualitative difference when RAGE TG mice and controls were compared (Figure 2A and B). Immunohistochemistry was subsequently performed for collagen IV, a prolific collagen subtype common in basement membrane structure. Qualitatively, staining for collagen IV revealed a discernable decrease in lungs from RAGE TG mice (Figure 2D) compared to control lung sections (Figure 2C). In order to quantitatively assess collagen IV abundance, immunoblotting using equal concentrations of lung homogenates was performed and collagen IV was significantly diminished in RAGE TG lungs compared to controls (Figure 2E).

**Figure 3:**
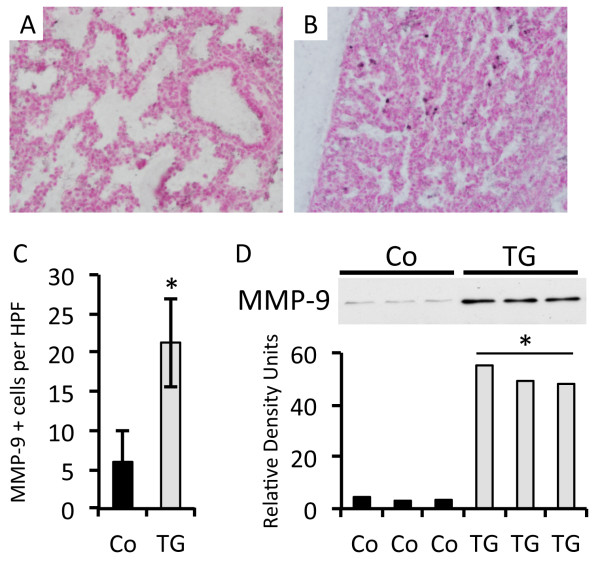
**MMP-9 expression in RAGE TG compared to controls.** Lungs from control **(**Co, **A)** and RAGE TG **(B)** mice were qualitatively immunostained for MMP-9 and blinded counts of MMP-9 positive (+) cells were averaged per high powered field **(**HPF, **C)**. Images were at 100X original magnification. Immunoblotting for MMP-9 using standardized aliquots of 10 μg total lung protein revealed markedly increased MMP-9 expression in RAGE TG mouse lungs compared to controls **(D)**. *Statistical difference (P ≤ 0.05) with at least three replicates per group.

In order to decipher functional molecules in possible mechanisms of collagen metabolism, an analysis of matrix metalloprotease 9 (MMP-9) was conducted. MMP-9 has recently been identified as a molecule downstream of RAGE signaling in the lung [16,17]. Immunostaining for MMP-9 revealed a marked increase in cells from RAGE TG mouse lung that were actively synthesizing the protease (Figure 3A and B). While MMP-9 positive cells were observed in all lung sections, cell counts per high powered field (HPF) revealed a significant increase in the lungs of RAGE TG mice compared to controls (Figure 3C). In order to quantitatively assess MMP-9 expression in lung homogenates, immunoblotting for MMP-9 was performed and band densities from RAGE TG mouse lungs coincided with the significant elevation of MMP-9 positive cells (Figure 3D).

### FoxM1 and endothelial cell abundance is decreased in RAGE over-expressing lungs

**Figure 4:**
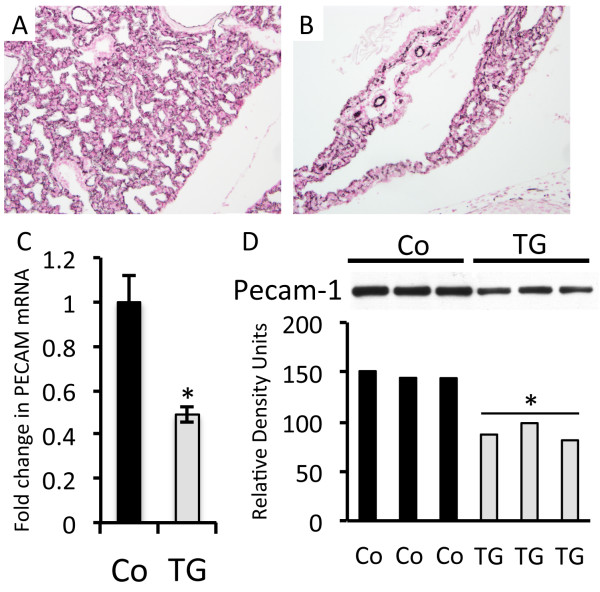
**Pecam-1 expression in RAGE TG compared to controls.** Lungs from control **(**Co, **A)** and RAGE TG **(B)** mice were qualitatively immunostained for Pecam-1, revealing no appreciable differences. Images were at 100X original magnification. Quantitative RT-PCR **(C)** and immunoblotting **(D)** for Pecam-1 using standardized aliquots of total lung mRNA or protein revealed markedly decreased Pecam-1 expression in RAGE TG mouse lung compared to controls. *Statistical difference (P ≤ 0.05) with at least three replicates per group.

Because lungs from RAGE TG mice exposed to dox from conception to E18.5 experienced significant epithelial cell death [5] and compromised basement membrane stability, endothelial cell biology was also considered due to its proximity. It was hypothesized that abnormalities in alveolar epithelial cell turnover might occur in tandem with the contraction of endothelial cells that share the same weakened basement membrane. Immunostaining for Pecam-1, a common endothelial cell marker, revealed indistinguishable differences when comparing RAGE TG and control lungs (Figure 4A and B). However, quantitative real-time PCR using equal concentrations of mRNA from both groups revealed a significant decrease in the expression of *Pecam-1* mRNA in RAGE TG mice (Figure 4C). The decrease in message was corroborated with immunoblotting for Pecam-1 in lung homogenates wherein protein quantification was significantly decreased in the lungs of RAGE TG mice compared to controls (Figure 4D).

**Figure 5:**
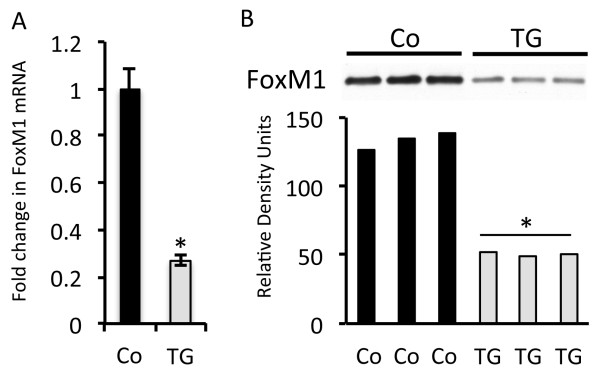
**FoxM1 expression in RAGE TG compared to controls.** Quantitative RT-PCR **(A)** and immunoblotting **(B)**for FoxM1 using standardized aliquots of total lung mRNA or protein revealed markedly decreased FoxM1 expression in RAGE TG mouse lung compared to controls (Co). *Statistical difference (P ≤ 0.05) with at least three replicates per group.

Because transcription factors that orchestrate epithelial cell differentiation were already known to be impaired in RAGE TG mice [10], an interest in transcriptional regulation and the derivation of endothelial cells was cultivated. FoxM1 was identified based on its effects on cell cycle progression in mesenchymal cells and because FoxM1 null mice exhibit pulmonary microvascular abnormalities associated with diminished Pecam-1 and vascular endothelial growth factor (VEGF) expression [18]. Real-time PCR analysis of *FoxM1* revealed a nearly three-fold decrease in the expression of the transcription factor in the lungs of RAGE TG mice compared to controls (Figure 5A). Furthermore, when comparing control lung samples, immunoblotting identified a significant decrease in the abundance of FoxM1 protein in RAGE TG lungs (Figure 5B).

## Discussion and conclusions

### Effects of RAGE over-expression on basement membrane

The use of a conditional mouse model involving alveolar epithelium allowed for the assessment of RAGE over-expression on the respiratory membrane that is comprised of alveolar epithelium, endothelium, and a fused basement membrane. A sound respiratory membrane in advanced organisms is critical to terrestrial life and accordingly, its strength and permeability must be maintained. A significant determinant of the basement membrane’s interposition between cells in the respiratory membrane is due to intricate collagen IV deposition [19,20]. In fact, the importance of collagen IV as a chief component of the blood-gas barrier is confirmed in research that identifies impaired basement membranes in clinical conditions including COPD [21]. Our qualitative and quantitative data revealing diminished collagen IV identifies an intriguing paradigm in which increased expression of pattern recognition receptors such as RAGE by epithelial cells can signal changes in the surrounding matrix.

RAGE-ligand interactions initiate signaling via the activation of signaling intermediates such as Ras, extracellular signal regulated kinase (ERK) 1/2, and c-Jun-NH2-terminal kinase (JNK) 1/2 prior to nuclear translocation of active NF-κB [22]. The current research sought to assess possible roles for MMP-9, a matrix metalloprotease (MMP) secreted by fibroblasts, alveolar macrophages, and epithelial cells through pathways culminating in NF-kB activation [23]. MMPs comprise a family of 24 related endopeptidases that degrade a variety of substrates, including components of the extracellular matrix, and MMP-9 specifically targets collagen IV [24]. The destructive functions of MMPs are critical in tissue remodeling observed in development and disease [25]. Based on their multiple biological activities, MMPs are considered participants in diverse pathological processes in the lung [26], but also as effectors during lung morphogenesis [27,28]. Notably, MMP-9 has been directly implicated in the progression of bronchopulmonary dysplasia (BPD), a developmental anomaly characterized by inflammation, lack of alveolar septation, and abnormal pulmonary vascular development [29]. We have shown that the over-expression of RAGE results in increased MMP-9 and decreased collagen IV. As recently demonstrated in adult RAGE TG mice [17], this particular study confirms that MMP-9 is increased via a RAGE-mediated pathway during development as well. While some additional studies in abdominal aortic aneurisms and HaCaT keratinocytes describe MMP-9 induction via RAGE signaling, only in the current study was lung development considered [30,31]. Our new evidence points to MMP-9-mediated remodeling in the developing lung under the control of RAGE signaling, a particularly important concept given the abundance of RAGE in the lung.

### Effects of RAGE over-expression on endothelium

Over-expression of RAGE by alveolar epithelial cells also directly influenced endothelial cell behaviors in the lung. The lung mesenchyme undergoes vasculogenesis (*de novo* formation of vessels) and angiogenesis (branching of new vessels from preexisting ones) in precisely controlled pathways that require appropriate levels of VEGF and other regulatory molecules [32,33]. We demonstrated that RAGE over-expression led to decreased development of pulmonary microvasculature associated with diminished levels of Pecam-1 [34]. Previous research revealed abnormal thyroid transcription factor-1 (*TTF-1*) expression in the lung of perinatal RAGE TG mice [10]. *TTF-1* is a phosphorylated member of the Nkx2 family of homeodomain-containing transcription factors and TTF-1 phosphorylation mutants have deficits in lung vascularity due to decreased Pecam-1 and VEGF expression [35]. A supplementary figure demonstrates significantly decreased TTF-1 expression in the lungs of RAGE TG mice compared to controls during periods through E18.5 [see Additional file 1: Figure S1]. It is therefore likely that decreased staining for Pecam-1 relates to misregulation of TTF-1 among other important factors.

A central feature likely involved in endothelial cell derivation and the coordination of vessel formation in the RAGE TG mouse lung are the contributions of FoxM1. The Forkhead Box (Fox) proteins consist of extensive transcription factors homologous with the Winged Helix/*Forkhead* DNA binding factors [36]. Notably, Fox proteins FoxA2, FoxJ1, FoxF1, and FoxP each play critical roles in transcriptionally regulating branching morphogenesis and vasculogenesis in the developing lung [37-39]. In particular, deletion of FoxM1 is lethal during the embryonic period and selective inhibition revealed it is important during pulmonary development where it activates genes involved in G1/S and G2/M progression through its interaction with Cdk-Cyclin complexes and p300/CBP coactivators [40]. Pulmonary microvascular abnormalities associated with diminished levels of Pecam-1, transforming growth factor (TGF)-β receptor type II, ADAM-17, VEGF receptors, FLK-1, Aurora B kinase, LAMA4, and the FoxF1 transcription factor were observed in lungs of FoxM1 null mice [18]. Conversely, conditional up-regulation of FoxM1 caused increased proliferation of alveolar and bronchial epithelial cells, as well as smooth muscle and endothelial cells via the activation of cell cycle promoting factors [41]. Because RAGE TG mice express less FoxM1, and VEGF is a direct transcriptional target of FoxM1 [42], the data identify likely impairment of genetic regulation of cell proliferation and extracellular matrix remodeling processes.

FoxM1 has also been centrally implicated in repair mechanisms following damage to epithelial and endothelial cell populations in the respiratory region of the lung. For example, FoxM1 was increased after LPS-induced vascular injury and FoxM1 participation in endothelial cell repair following injury has been suggested [43]. A prospective mechanism for FoxM1-mediated repair centers on β-catenin, a protein that forms adherens junctions between neighboring endothelial cells. Mirza and others have clearly shown that FoxM1 is a key player in the re-establishment of the alveolar blood-gas barrier following injury via the restoration of β-catenin in endothelial cell junctions after vascular injury [44]. On the opposite face of the respiratory membrane, alveolar epithelial cells increase FoxM1 expression following butylated hydroxytoluene (BHT)-induced lung injury [45] and FoxM1 is linked to the coordination of enhanced ATII-ATI cell transition following cellular damage [46]. The general theme of FoxM1-mediated protection from apoptosis has also been shown in cancer cells [47] and is therefore an important regulator of homeostasis in the context of both repair and cell turnover.

RAGE over-expression and/or ligand-induced over-activation may underpin the early development of lung disease states such as perinatal BPD and emphysema in the adult lung. Our work has previously shown that tobacco smoke rich in AGEs enhance RAGE expression and signaling [22]. The data presented here compliment early work by Voelkel and colleagues that showed enhanced endothelial cell apoptosis as an early feature of smoke-induced emphysema [48]. More recently, Gordon et al. have shown that endothelial particles are observed as a precursor to measurable lung destruction in smokers [49]. Accordingly, in addition to disease progression observed in mature lungs, this work further implicates RAGE signaling in pathways that regulate lung remodeling during embryogenesis.

In summary, matrix destabilization and FoxM1-mediated effects on respiratory membrane cells are probable determinants of the hypoplastic lung observed in perinatal RAGE TG mice. Our prior research that revealed a fas ligand-mediated shift toward apoptosis in the lung periphery [5] and impaired ATII-ATI transition reveals that both abnormal apoptosis and loss of repair mechanisms mediated by FoxM1 likely result in impaired lung development. Even with these important advancements in the study of RAGE signaling during lung morphogenesis, additional research is still required that centers on precise signaling pathways involved in cell responses to RAGE up-regulation.

## Competing interests

The authors declare that they have no competing interests.

## Authors’ contributions

NTF, BRB, AJG, ABR, and JAS performed immunohistochemistry and AJW and ZRJ assisted with immunoblotting. NTF and ABR were responsible for the qRT-PCR experiments. PRR conceived of the study and with the assistance of DRW, supervised in its implementation, interpretation, and writing. All authors assisted in manuscript preparation and approved of the final submitted version.

## Supplementary Material

Additional file 1: Figure S1 A. Lung homogenates from E15.5-E18.5 RAGE TG mice and controls were immunoblotted for TTF-1 for using a rabbit polyclonal antibody (WRAB-76694, Seven Hills Boireagents, Cincinnati, OH) at a dilution of 1:1000. B. Densitometry of resulting bands were quantified as outlined in the Methods. Click here for file
