# Supplementary figures and images for: Conditional over-expression of RAGE by embryonic alveolar epithelium compromises the respiratory membrane and impairs endothelial cell differentiation
Source: Respir Res. 2013 Oct 17;14(1):108. doi: 10.1186/1465-9921-14-108 (PMC3853184; doi:10.1186/1465-9921-14-108)

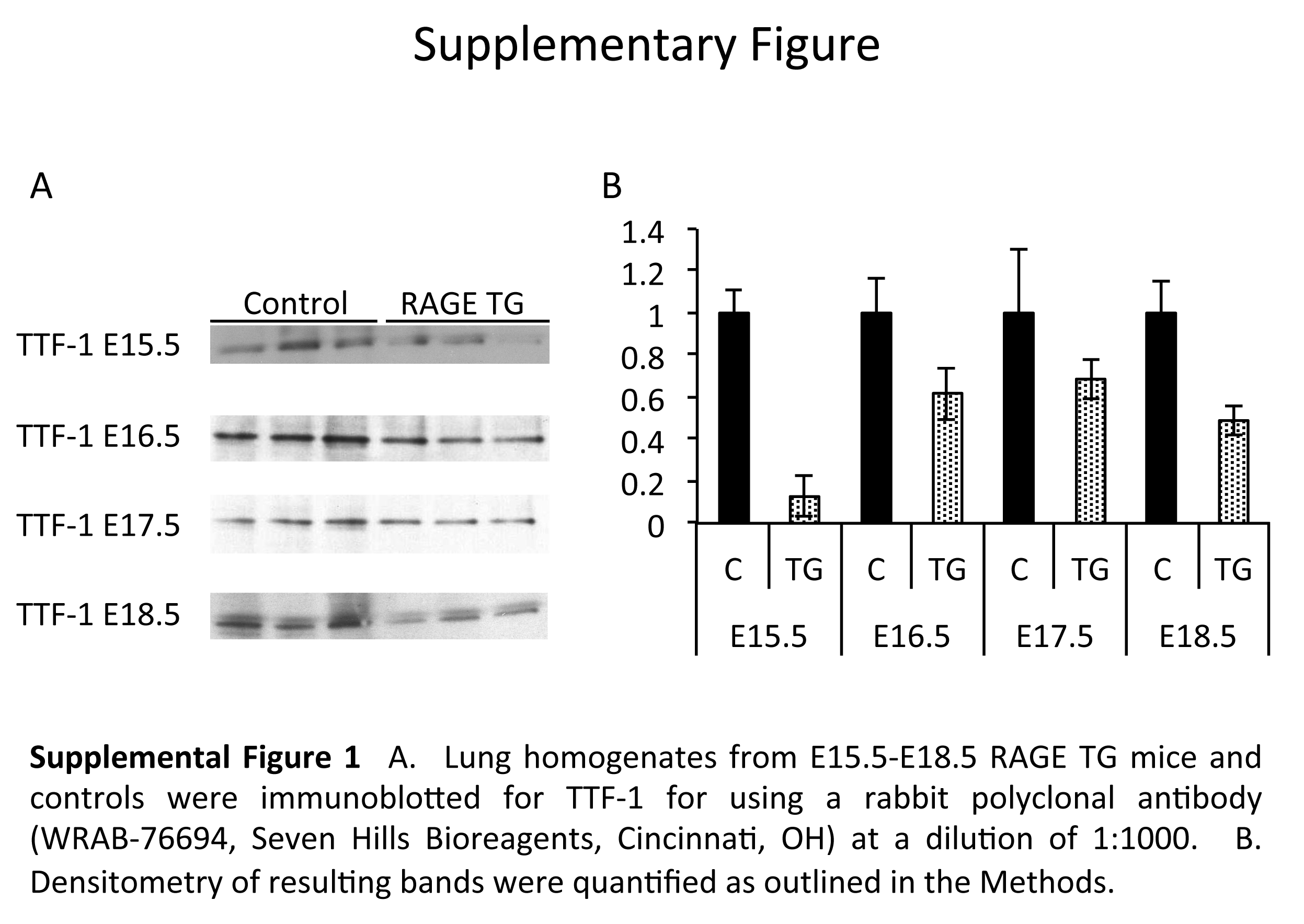

Supplement: Additional file 1: Figure S1 — A. Lung homogenates from E15.5-E18.5 RAGE TG mice and controls were immunoblotted for TTF-1 for using a rabbit polyclonal antibody (WRAB-76694, Seven Hills Boireagents, Cincinnati, OH) at a dilution of 1:1000. B. Densitometry of resulting bands were quantified as outlined in the Methods. [file 1465-9921-14-108-S1.tiff]
